# Prognostication and Risk Factors for Cystic Fibrosis via Automated Machine Learning

**DOI:** 10.1038/s41598-018-29523-2

**Published:** 2018-07-26

**Authors:** Ahmed M. Alaa, Mihaela van der Schaar

**Affiliations:** 1 Department of Electrical Engineering, University of California, Los Angeles, CA 90095 USA; 2 Alan Turing Institute, London, NW1 2DB UK; 3 Engineering Science Department, University of Oxford, Oxford, OX1 3PJ UK

## Abstract

Accurate prediction of survival for cystic fibrosis (CF) patients is instrumental in establishing the optimal timing for referring patients with terminal respiratory failure for lung transplantation (LT). Current practice considers referring patients for LT evaluation once the forced expiratory volume (FEV_1_) drops below 30% of its predicted nominal value. While FEV_1_ is indeed a strong predictor of CF-related mortality, we hypothesized that the survival behavior of CF patients exhibits a lot more heterogeneity. To this end, we developed an algorithmic framework, which we call AutoPrognosis, that leverages the power of machine learning to automate the process of constructing clinical prognostic models, and used it to build a prognostic model for CF using data from a contemporary cohort that involved 99% of the CF population in the UK. AutoPrognosis uses Bayesian optimization techniques to automate the process of configuring ensembles of machine learning pipelines, which involve imputation, feature processing, classification and calibration algorithms. Because it is automated, it can be used by clinical researchers to build prognostic models without the need for in-depth knowledge of machine learning. Our experiments revealed that the accuracy of the model learned by AutoPrognosis is superior to that of existing guidelines and other competing models.

## Introduction

Cystic fibrosis (CF) is an autosomal recessive disease caused by the presence of mutations in both alleles at the cystic fibrosis transmembrane conductance regulator (CFTR) gene, and is the most common genetic disease in Caucasian populations^1,2^. Impaired CFTR functionality gives rise to different forms of lung dysfunction, all of which eventually lead to progressive respiratory failure^3,4^. Despite recent therapeutic progress that significantly improved CF prognosis^5^, only half of the current CF population are expected to live to over 40 years old^6^. Lung transplantation (LT) is recommended for patients with end-stage respiratory failure as a means to improved life expectancy^7–9^. Unfortunately, there are more LT candidates than available lung donors^7^, and in addition, the LT procedure is accompanied by serious risks of subsequent post-transplant complications^10^. An effective LT referral policy should ensure an efficient allocation of the scarce donor lungs by precisely identifying high-risk patients as candidates for transplant, without overwhelming the LT waiting list with low-risk patients for whom a LT might be an unnecessary exposure to the risk of post-transplant complications^11^. The goal of this paper is to develop a CF prognostic model that can guide clinical decision-making by precisely selecting high-risk patients for LT referral.

Current consensus guidelines, such as those recommended by the International Society for Heart and Lung Transplantation (ISHLT)^12^, consider referring a patient for LT evaluation when the forced expiratory volume (FEV_1_) drops below 30% of its predicted nominal value. This guideline, which is widely followed in clinical practice^13,14^, is based mainly on the seminal study by Kerem *et al*.^15^, which identified FEV_1_ as the main predictor of mortality in CF patients using survival data from a cohort of Canadian CF patients (patients eligible 1977–1989). While the FEV_1_ biomarker has been repeatedly confirmed to be a strong predictor of mortality in CF patients^10,16,17^, recent studies have shown that the survival behavior of CF patients with FEV_1_ < 30% exhibits substantial heterogeneity^18^, and that the improvements in CF prognosis over the past years have changed the epidemiology and demography of CF populations^19,20^, which may have consequently altered the relevant CF risk factors (A striking example of a significant change in the demography of the CF population is the sharp decline in pediatric mortality in recent years^19^). However, none of the existing prognostic models that combine multiple risk factors^21–24^ have been able to demonstrate a significant improvement in mortality prediction compared to the FEV_1_ criterion in terms of the positive predictive value, which is a proximal measure for the rate of premature LT referral (low-risk patients referred to a transplant)^10^.

In this paper, we leverage *machine learning* algorithms to discover an accurate, data-driven prognostic model and CF risk factors on the basis of a contemporary cohort from the UK CF registry; a database that includes 99% of the CF population in the UK^25–27^. While machine learning has proven successful in providing high predictive accuracies in clinical settings with heterogeneous populations^28^, its deployment in healthcare research and practice has been limited (e.g. only 15% of hospitals in the US use machine learning only for rather limited purposes^29^). A main hindrance to wide deployment of machine learning in clinical research is the need for the in-depth expertise that is necessary for making complex design choices on what algorithm to use and how to tune the algorithm’s hyper-parameters^29^. We would like to have a machine learning framework that is easily accessible by clinicians and CF centers. In addition, because the CF population demography, epidemiology and therapeutic options are evolving rapidly, we would like a prognostic model that can be updated and re-calibrated annually in an automated fashion whenever data from the most recent annual review becomes available in the registry.

**Figure 1:**
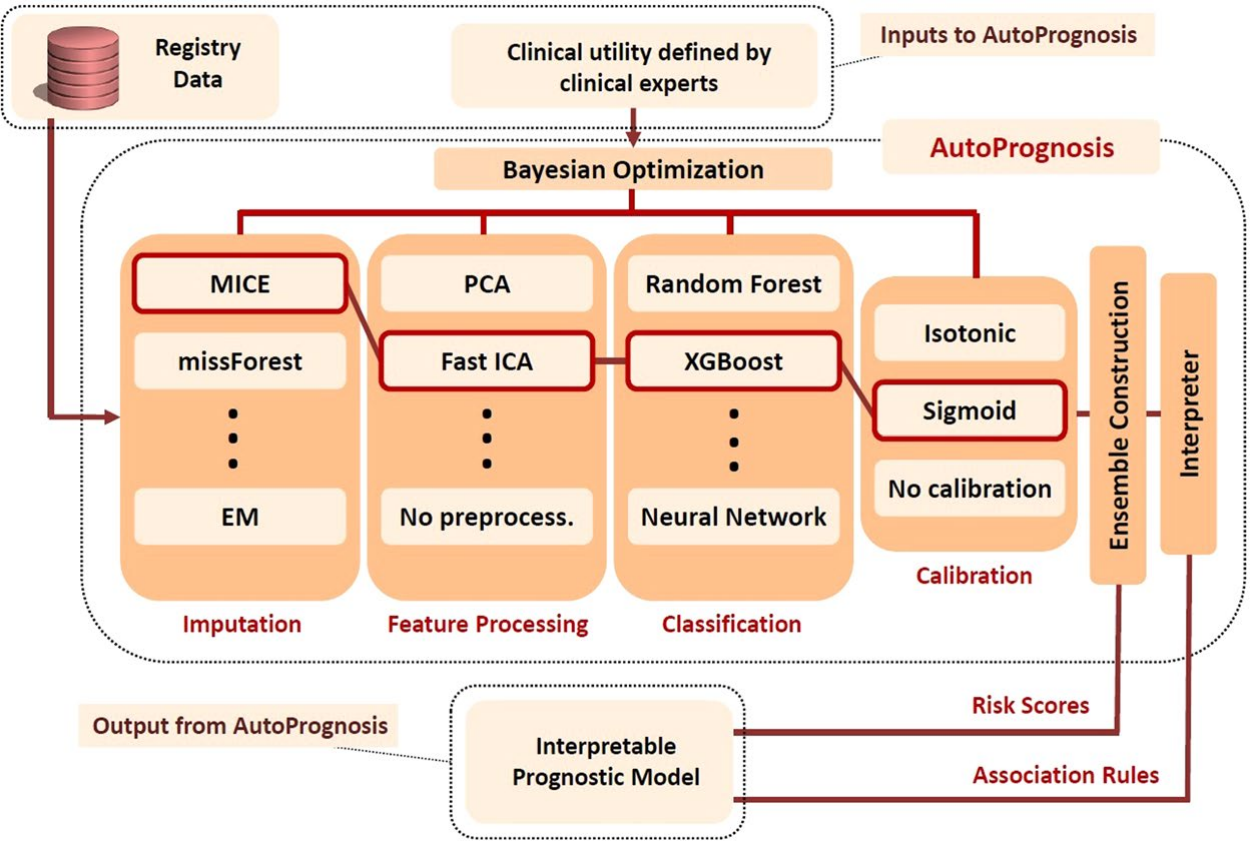
Schematic depiction of the AutoPrognosis framework. AutoPrognosis is provided with a dataset and a definition for an appropriate clinical utility selected by clinical experts. The algorithm uses Bayesian optimization in order to update its beliefs about the clinical utility of different machine learning pipelines, where each pipeline comprises an imputation algorithm, a feature processing algorithm, a classification algorithm and a calibration method. In this depiction, a pipeline comprising MICE imputation, fast ICA processing, XGBoost classifier and sigmoid calibration is highlighted.

In order to fully exploit the potentiality of machine learning in CF prognostication, we developed an algorithmic framework and a software package, dubbed *AutoPrognosis*, which adopts an automated machine learning (AutoML)^30^ approach for constructing optimized clinical prognostic models. An overview of the AutoPrognosis framework is provided in Fig. 1. AutoPrognosis uses Bayesian optimization techniques^31^ in order to (efficiently) identify the machine learning *pipelines* (out of a huge space of possible pipelines) that maximize a predefined diagnostic accuracy metric, where a pipeline consists of an imputation algorithm, a feature processing algorithm, a classification algorithm and a calibration method. AutoPrognosis combines the best performing pipelines in a single, well-calibrated predictive ensemble by weighting the different pipelines using the algorithm’s posterior belief about each pipeline’s clinical utility. The AutoPrognosis framework is currently implemented as a Python module, and it supports 7 imputation algorithms, 14 feature processing algorithms, 20 classification algorithms, and 3 calibration methods; a design space which corresponds to a total of 5,880 pipelines (The software implementation of AutoPrognosis can be very easily updated with more algorithmic components over time.) The Bayesian optimization algorithm used by AutoPrognosis implements a sequential exploration-exploitation scheme in which balance is achieved between exploring the clinical utility of new pipelines and re-examining the utility of previously explored ones^32^, where the clinical utility is predefined by clinical researchers as a (cost-sensitive) function of the achieved diagnostic accuracy. Our adoption of a Bayesian optimization framework is motivated by its recent remarkable success in optimizing black-box functions with costly evaluations as compared to simpler approaches such as grid and random search^32^. The final stage of AutoPrognosis is an “ *interpreter*” module, which uses an *associative classifier*^33,34^ to explain the predictions of the black-box prognostic ensemble learned by the preceding stage, allowing for prognostic model interpretability without degrading the predictive performance. Detailed explanation for the components and operation of AutoPrognosis is provided in Methods. A technically-oriented report on our system can be found in Alaa *et al*.^35^.

We conducted an extensive analysis of the performance of AutoPrognosis, and compared it to those achieved by the existing guidelines, competing clinical models and other machine learning algorithms. Our analysis shows that AutoPrognosis displays clear superiority to all competing methods in terms of both diagnostic accuracy and impact on clinical decision-making. In particular, AutoPrognosis was capable of achieving a positive predictive value of 65% (95% CI: 61–69%), whereas that achieved by the FEV_1_ criterion recommended by the guidelines is as low as 48% (95% CI: 44–52%), at a fixed sensitivity level. To the best of our knowledge, this is the best reported reduction in premature LT referrals since the seminal study by Mayer-Hamblett *et al*.^10^. While the vast majority of clinical literature has focused on spirometric biomarkers reflecting airway obstruction as the main CF risk factors, AutoPrognosis revealed new insight on the importance of variables reflecting disorders in pulmonary gas exchange in improving the precision and clinical usefulness of prognostic models. AutoPrognosis was also able to identify moderate-risk patient groups that experience intermediate adverse outcomes such as short-term pulmonary decline.

We envision AutoPrognosis as being embedded in a computerized support system that is fed annually with the most recent CF review data, providing clinical researchers and CF centers with up-to-date prognostic models, new risk factors, and explanations for risk predictions. While we focus in this study on CF prognostication, the AutoPrognosis framework can be applied to construct prognostic models for any other disease.

## Results

### Data and experimental setup

Experiments were conducted using retrospective longitudinal data from the UK cystic fibrosis Registry; a database sponsored and hosted by the UK cystic fibrosis Trust^25^. The registry comprises a list of annual follow-up variables for individual CF patients that includes demographics, genetic mutations, airway colonization and microbiological infections, comorbidities and complications, transplantation, hospitalization, spirometry and therapeutic management. We used AutoPrognosis to automatically construct a prognostic model for predicting 3-year mortality (a realistic waiting time in a lung transplantation waiting list^10^) based on the follow-up variables at baseline.

**Figure 2:**
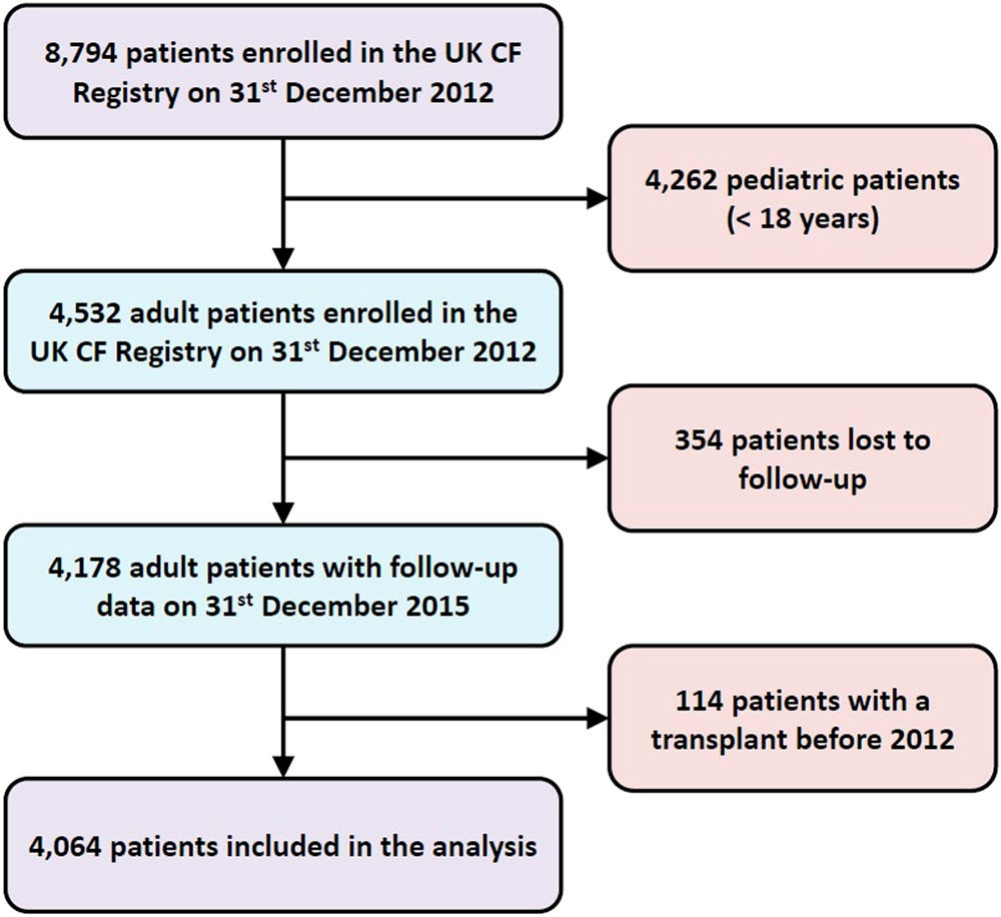
Patient selection and data assembly process.

All experiments were conducted using data for a baseline cohort comprising patients’ follow-up variables collected in 2012: this was the most recent cohort for which 3-year mortality data was available. A total of 115 variables were associated with every patient, all of which were fed into AutoPrognosis in order to encourage an agnostic, data-driven approach for discovering risk factors. Since transplantation decisions are mostly relevant for adults (93.75% of transplantation operations recorded in the registry were performed in adults), we excluded pediatric patients, and included only patients who were more than 18 years old. (Deaths in children with CF are now very rare in developed countries^19,36^). Outcomes are defined as death or lung transplantation within 3 years of the baseline data collection date. Patients who were lost to follow-up or have already undergone a transplant before 2012 were excluded. Figure 2 depicts a flow chart of the data assembly process involved in our analysis. Of the 4,532 patients who were aged 18 years or older in 2012, a total of 114 patients underwent a lung transplant before their 2012 annual review, and a total of 354 patients were lost to follow-up. Of the remaining 4,064 patients, 382 patients (9.4%) experienced an adverse outcome within a 3-year period.

Of the 382 patients who experienced an adverse outcome, 266 died without receiving a transplant, 104 underwent a successful transplant, and 12 patients received a transplant but died within the 3-year horizon. We incorporated each patient’s spirometric trajectory by extracting the FEV_1_% predicted for all patients in the years 2008 to 2011. The characteristics of the patients in the baseline cohort are provided in Table 1. The study population was stratified into two subgroups based on the endpoint outcomes and the characteristics of the two subgroups were compared using Fisher’s exact test for discrete (and categorical) variables, and Mann-Whitney *U* test for continuous variables. The number of CFTR mutations (in either alleles) whose frequencies in the cohort exceeded 1% was 66, with the most frequent five mutations being ΔF508, G551D, R117H, G542X, and 621 + 1G → T. Previous studies on CF genetics have classified CFTR mutations into 6 different categories according to the mechanism by which they obstruct the synthesis and traffic of CFTR^2^. We used the CFTR genetic classification in order to cluster the (high-dimensional) genotype information. In particular, we converted the genotype information of every patient into a vector of 9 binary features which encodes the following information: whether the CFTR mutation is homozygous, whether any of the two alleles carries a ΔF508 or a G551D mutation, and the class to which the mutation carried by the patient belongs. All the encoded genetic features are listed in Table 1. Examples for the mutations and molecular consequences^37^ of every class are provided in Table 2.

**Table 1:** Baseline characteristics of patients in the UK CF Registry on December 31st 2012. (^§^Continuous variables: median (inter-quartile range)).

Variable	Alive & no LT n = 3,682 (%)	Death/LT n = 382 (%)	*p*-value	Variable	Alive & no LT n = 3,682 (%)	Death/LT n = 382 (%)	*p*-value
**Gender** (**% male**)	2,027 (55.0)	192 (50.2)	0.075	*Pancreatic*			
**Age** (**years**)^**§**^	27.6 (12)	29.2 (14)	<0.001	Cirrhosis	86 (2.3)	24 (6.3)	<0.001
**Height** (**cm**)^**§**^	168.0 (14)	166.0 (15)	<0.001	Liver Disease	578 (15.7)	81 (21.2)	0.007
**Weight** (**kg**)^**§**^	63.1 (17)	54.8 (15)	<0.001	Pancreatitis	57 (1.5)	3 (0.8)	0.368
**BMI** (**kg/m**^**2**^)^**§**^	22.3 (4)	20.1 (4)	<0.001	Liver Enzymes	521 (14.1)	98 (25.7)	<0.001
**CFTR genotype**				Gall Bladder	20 (0.5)	3 (0.8)	0.472
Homozygous	1,784 (48.4)	208 (54.4)	<0.001	GI Bleed (variceal)	3 (0.1)	3 (0.8)	0.013
Heterozygous	1,240 (33.7)	92 (24.0)	<0.001	*Gastrointestinal*			
ΔF508	3,189 (86.6)	325 (85.0)	0.388	GERD	747 (20.3)	100 (26.2)	0.008
G551D	224 (6.0)	15 (3.9)	0.108	GI Bleed (no variceal)	4 (0.1)	1 (0.3)	0.390
Class I	169 (4.6)	23 (6.0)	0.205	Intestinal Obstruction	303 (8.2)	33 (8.6)	0.770
Class II	3,207 (87.1)	326 (85.3)	0.338	*Musculoskeletal*			
Class III	3,281 (89.1)	330 (86.3)	0.123	Arthropathy	338 (9.2)	52 (13.6)	0.008
Class IV	184 (5.0)	4 (1.0)	<0.001	Bone Fracture	39 (1.1)	6 (1.6)	0.310
Class V	130 (3.5)	8 (2.0)	0.179	Osteopenia	710 (19.3)	126 (33.0)	<0.001
Class VI	3,189 (86.6)	325 (85.0)	0.388	*Other*			
**Spirometry** ^**§**^				Cancer	8 (0.2)	5 (1.3)	0.005
FEV_1_ (L)	2.34 (1.4)	0.99 (0.6)	<0.001	Diabetes	906 (24.6)	199 (52.1)	<0.001
FEV_1_%	67.8 (35)	29.6 (19)	<0.001	CFRD	1,096 (29.8)	223 (58.4)	<0.001
Best FEV_1_ (L)	2.57 (1.4)	1.2 (0.7)	<0.001	Pulmonary Abscess	2 (0.1)	0 (0.0)	1.000
Best FEV_1_%	75.2 (31)	35.2 (18)	<0.001	Chronic Pseudomonas	2,005 (54.5)	281 (73.6)	<0.001
FEV_1_% (2011)	70.2 (34)	36.2 (20)	<0.001	Osteoporosis	293 (8.0)	91 (23.8)	<0.001
FEV_1_% (2010)	70.7 (33)	37.5 (23)	<0.001	AICU	5 (0.1)	1 (0.3)	0.447
FEV_1_% (2009)	72.2 (32)	41.2 (27)	<0.001	Kidney Stones	45 (1.2)	17 (4.5)	<0.001
FEV_1_% (2008)	73.7 (31)	45.2 (27)	<0.001	Cough Fracture	1 (0.0)	3 (0.8)	0.003
**Lung Infections**				Hypertension	121 (3.3)	23 (6.0)	0.012
B. Cepacia	176 (4.8)	35 (9.2)	0.001	Atypical Mycobacteria	127 (3.4)	17 (4.5)	0.308
P. Aeruginosa	2,190 (59.5)	295 (77.2)	<0.001	Hearing Loss	82 (2.2)	26 (6.8)	<0.001
MRSA	154 (4.2)	17 (4.5)	0.789	Depression	257 (7.0)	59 (15.4)	<0.001
Aspergillus	478 (13.0)	70 (18.3)	0.006	**Inhaled Antibiotics**	2,194 (59.6)	280 (73.3)	<0.001
NTM	186 (5.1)	20 (5.2)	0.902	**Muco-active Therapies**			
H. Influenza	191 (5.2)	10 (2.6)	0.025	DNase	2,057 (55.9)	297 (77.7)	<0.001
E. Coli	17 (0.5)	2 (0.5)	0.698	Hypertonic Saline	859 (23.3)	109 (28.5)	0.027
K. Pneumoniae	10 (0.3)	3 (0.8)	0.116	**Promixin**	765 (20.8)	71 (18.6)	0.352
Gram-negative	14 (0.4)	4 (1.0)	0.082	**Tobramycin**	110 (3.0)	28 (7.3)	<0.001
ALCA	97 (2.6)	25 (6.5)	<0.001	**iBuprofen**	8 (0.2)	2 (0.5)	0.241
Staph. Aureus	1,175 (31.9)	64 (16.8)	<0.001	**Oral Corticosteroids**	347 (9.4)	122 (31.9)	<0.001
Xanthomonas	165 (4.5)	23 (6.0)	0.199	**IV Antibiotics**	1493 (40.5)	292 (76.4)	<0.001
B. Multivorans	86 (2.3)	16 (4.2)	0.037	**IV Antibiotic Courses** ^**§**^			
B. Cenocepacia	51 (1.4)	13 (3.4)	0.007	Days at Home	0 (14)	14 (49)	<0.001
Pandoravirus	8 (0.2)	2 (0.5)	0.241	Days at Hospital	0 (13)	27.5 (56)	<0.001
**Comorbidities**				**Non-IV Hospitalization**	312 (8.5)	62 (16.2)	<0.001
*Respiratory*				**Non-invasive Ventilation**	161 (4.4)	82 (21.5)	<0.001
ABPA	432 (11.7)	71 (18.6)	<0.001	**Oxygen Therapy**	279 (7.6)	205 (53.7)	<0.001
Nasal Polyps	123 (3.3)	4 (1.0)	0.012	Continuous	13 (0.4)	75 (19.6)	<0.001
Asthma	578 (15.7)	58 (15.2)	0.825	Nocturnal	42 (1.1)	48 (12.6)	<0.001
Sinus Disease	486 (13.2)	41 (10.7)	0.200	Exacerbation	100 (2.7)	46 (12.0)	<0.001
Hemoptysis	48 (1.3)	11 (2.9)	0.022	Pro re nata	37 (1.0)	29 (7.6)	<0.001

**Table 2:** Exemplary mutations and molecular consequences of the 6 genetic classes.

Genetic class	Molecular consequence
Class I	No functional protein produced
Class II	Diminished protein processing
Class III	Defective gating
Class IV	Decreased conductance
Class V	Abnormal splicing
Class VI	Decreased cell surface stability
**Genetic class**	**Mutations**
Class I	G542X, W1282X, 1717-1G → A
Class II	N1303K, ΔF508, A455E
Class III	G551D, R117H, ΔF508
Class IV	R117H, R347H, R347P, R334W
Class V	621 + 1G → T, 3849 + 10kbC → T, 2789 + 5G → A
Class VI	ΔF508

### Training and validation of AutoPrognosis

**Figure 3:**
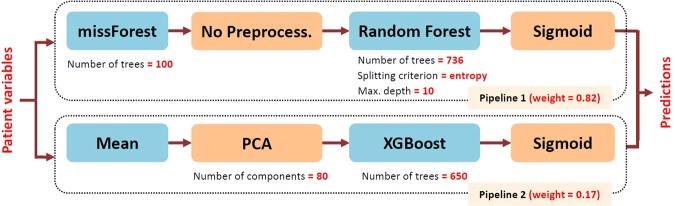
Schematic depiction for the in-sample model fit obtained by AutoPrognosis.

**Figure 4:**
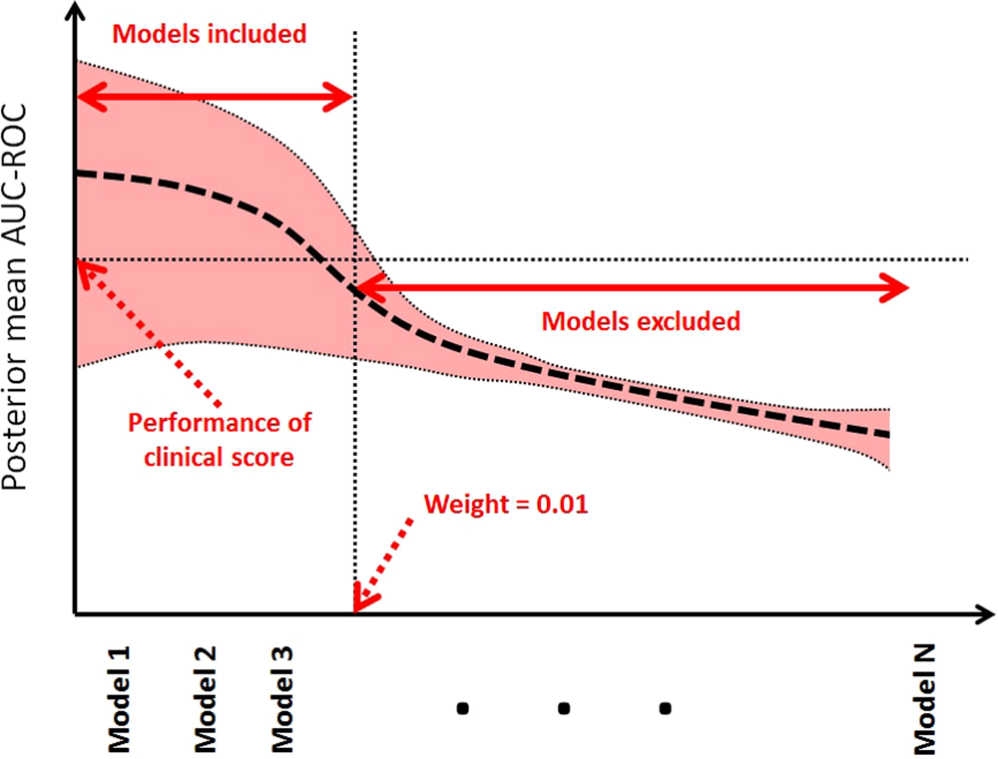
Depiction of the criterion for pipeline inclusion in the final AutoPrognosis ensemble.

All evaluations of diagnostic accuracy in the following subsections were obtained via 10-fold stratified cross-validation in order to assess the generalization performance, where a held-out sample was used to evaluate the performance of the model learned by AutoPrognosis in every fold using a mutually exclusive training sample. In every cross-validation fold, AutoPrognosis conducts up to 200 iterations of a Bayesian optimization procedure (details are provided in Methods), where in every iteration it explores a new pipeline and tunes its hyper-parameters. AutoPrognosis builds an ensemble of all the pipelines that it explored in which every pipeline is given a weight that is proportional to its empirical performance. All explored models with a posterior mean performance that is less than the best performance reported in the clinical literature (or equivalently, all models with weight less than 0.01 as shown in Fig. 4) were excluded from the final ensemble. The final model that would be used in actual practice is fit to the entire dataset; the pipeline configuration corresponding to the in-sample model fit obtained by AutoPrognosis is depicted in Fig. 3. The model combines two pipelines: the first uses *missForest* imputation^38^ and a *random forest* classifier (with 736 trees) with no feature processing, whereas the second pipeline uses simple mean imputation, a PCA transformation with 80 components followed by an XGBoost classifier with 650 trees. Both pipelines used sigmoid regression for calibration. The achieved in-sample area under receiver operating characteristic curve was 0.9714, and the model was well-calibrated, with a Brier score of 0.0543. The clinical utility function used for optimizing the prognostic model was the average of the area under the precision-recall curve and the average precision; the definitions of these metrics and the rationale behind using them as measures of the clinical utility will be clarified in the following subsections. The detailed training procedure is explained in Methods.

### Comparing AutoPrognosis with state-of-the-art prognostic models

#### Systematic review

We compared the diagnostic accuracy of AutoPrognosis with state-of-the-art prognostic models that were developed for predicting short-term CF outcomes. In order to identify and select the competing prognostic models, we searched PubMed for studies published in the last 10 years (in all languages) with the terms “(cystic fibrosis) and survival and (prognostic or predictive model)”. We filtered the relevant studies by their clinical end-points, focusing only on studies that defined the composite end-point of death and lung transplantation in a time horizon of less than 5 years. We identified 3 contemporary studies that developed and validated prognostic models using multicenter or registry data^23,36,39,40^. In the first study, Buzzetti *et al*.^23^ developed a parsimonious multivariate logistic regression model for predicting 5-year outcomes for CF patients using 4 variables, and demonstrated that it outperforms the model developed by Liou *et al*.^22^ using retrospective data from 9 Italian CF centers. McCarthy *et al*.^39^ developed a predictive model, dubbed “CF-ABLE”, for predicting 4-year CF outcomes using 4 variables, and validated their model using data for 370 patients enrolled in the Irish CF registry data. Dimitrov *et al*.^40^ proposed a modified version of the CF-ABLE score, dubbed “CF-ABLE-UK”, which they (externally) validated through the UK CF registry data, reporting a c-statistic of 0.80 (95% CI: 0.79–0.83). More recently, Nkam *et al*.^36^ developed a multivariate logistic regression model for predicting 3-year CF outcomes using 8 risk factors. The model was internally validated through the French CF registry, reporting a c-statistic of 0.91 (95% CI: 0.89–0.92). We compared the diagnostic accuracy of AutoPrognosis with these 3 models as they considered similar clinical end-points and were validated on contemporary retrospective cohorts.

All of the studies mentioned above explored the usage of only a few risk factors in model development. To the best of our knowledge, ours is the first study to investigate an agnostic, machine learning-based approach for discovering risk factors for CF using a representative cohort that covers the entire CF population in the UK. In order to assess the clinical utility of AutoPrognosis, we also compared its diagnostic accuracy with the simple FEV_1_-based prediction rule proposed by Kerem *et al*.^15^, where a LT referral criterion that selects CF patients with an FEV_1_% of less than 30% predicted was recommended. This simple prediction rule continues to be the main criterion for LT referral in current clinical practice guidelines^13,14,41^.

#### Diagnostic accuracy evaluation

The main objective of CF prognostic models is to inform LT referral decisions^7,10,14,42^. Since donor lungs are scarce^7,8,11^, the clinical utility of a prognostic model should be quantified in terms of the model’s ability to (precisely) identify patients who are truly at risk and hence should be allocated in a LT waiting list. Many of the previously developed models have been validated only through goodness-of-fit measures^21,24^, which reveal little information about the models’ actual clinical utility. The area under receiver operating characteristic (AUC-ROC) curve has been used to quantify the discriminative power of the models developed by Nkam *et al*.^36^, McCarthy *et al*.^39^ and Buzzetti *et al*.^23^. AUC-ROC is nevertheless a misleading quantifier for the usefulness of a CF prognostic model as it is insensitive to the prevalence of poor outcomes in the population, and assumes that positive and negative predictions are equally important^43^. Since most patients would not need a LT at the 3-year horizon (the prevalence of poor outcomes is as low as 9.4%), a model’s AUC-ROC evaluation can be deceptively high, only reflecting a large number of “easy” and “non-actionable” true negative predictions, without reflecting the actual precision of the LT referral decisions guided by the model. The inappropriateness of AUC-ROC as a sole measure of diagnostic accuracy in the context of LT referral for CF patients was highlighted by Mayer-Hamblett *et al*.^10^, where it was shown that models with seemingly high AUC-ROC can still have modest predictive values (refer to Table 3 therein). A detailed technical analysis of the shortcomings of the AUC-ROC in imbalanced datasets was recently conducted by Saito *et al*.^44^.

**Table 3:** Comparison of various diagnostic accuracy metrics (with 95% CI) for the prognostic models under consideration.

Prognostic model	AUC-ROC	Youden’s J statistic	AUC-PR	Average Precision	*F*_1_ score
AutoPrognosis	0.89 ± 0.01	0.67 ± 0.02	0.58 ± 0.04	0.59 ± 0.04	0.60 ± 0.03
Nkam *et al*.^36^	0.86 ± 0.01	0.58 ± 0.03	0.50 ± 0.03	0.48 ± 0.03	0.52 ± 0.02
Buzzetti *et al*.^23^	0.83 ± 0.01	0.54 ± 0.03	0.42 ± 0.02	0.44 ± 0.03	0.49 ± 0.02
CF-ABLE-UK^40^	0.77 ± 0.01	0.48 ± 0.05	0.28 ± 0.04	0.20 ± 0.02	0.34 ± 0.02
FEV_1_% predicted criterion^15^	0.70 ± 0.01	0.41 ± 0.02	0.50 ± 0.02	0.27 ± 0.02	0.47 ± 0.01
SVM	0.84 ± 0.03	0.60 ± 0.05	0.50 ± 0.09	0.51 ± 0.09	0.52 ± 0.07
Gradient Boosting	0.87 ± 0.02	0.63 ± 0.01	0.55 ± 0.03	0.55 ± 0.04	0.56 ± 0.01
Bagging	0.83 ± 0.03	0.58 ± 0.05	0.51 ± 0.04	0.47 ± 0.04	0.52 ± 0.03
Pipeline 1 (grid search)	0.83 ± 0.02	0.56 ± 0.03	0.51 ± 0.04	0.47 ± 0.04	0.51 ± 0.03
Pipeline 1 (random search)	0.84 ± 0.01	0.56 ± 0.02	0.53 ± 0.02	0.49 ± 0.032	0.53 ± 0.02
Pipeline 2 (grid search)	0.87 ± 0.03	0.62 ± 0.02	0.54 ± 0.05	0.55 ± 0.03	0.57 ± 0.01
Pipeline 2 (random search)	0.83 ± 0.02	0.56 ± 0.03	0.51 ± 0.04	0.47 ± 0.04	0.51 ± 0.03
TPOT	0.84 ± 0.01	0.56 ± 0.03	0.51 ± 0.02	0.49 ± 0.02	0.51 ± 0.02

In order to ensure a comprehensive assessment for the clinical usefulness of AutoPrognosis, we evaluated the *positive predictive values* (PPV) and *negative predictive values* (NPV) for all predictive models under consideration, in addition to the standard AUC-ROC metrics. (PPV is also known as the *precision* metric.) The PPV reflects the fraction of patients who are truly at risk among those identified by the model as high risk patients. A model’s PPV characteristic best represents its clinical usefulness as it reflects the precision in the associated LT referral decisions^10^. That is, at a fixed sensitivity, models with higher PPV would lead to fewer patients who are not at risk being enrolled in a transplant waiting list, resulting in a more effective lung allocation scheme with fewer premature referrals.

In Table 3, we compare the performance of AutoPrognosis with the competing models in terms of various diagnostic accuracy metrics that capture the models’ sensitivity, specificity and predictive values. In particular, we evaluate the models’ AUC-ROC, Youden’s J statistic, area under precision-recall curve (AUC-PR), average precision and the *F*_1_ score. The AUC-ROC and Youden’s J statistic characterize the models’ sensitivity and specificity; the J statistic, also known as the *“informedness”*, characterizes the probability of an “informed decision”, and is computed by searching for the optimal cutoff point on the ROC curve that maximizes the sum of sensitivity and specificity^45,46^. As discussed earlier, the clinical usefulness of a model is better represented via its PPV characteristics, and hence we evaluate the models’ AUC-PR, average precision and *F*_1_ scores. The three metrics characterize the models’ precision (PPV) and recall (sensitivity): the AUC-PR is an estimate for the area under the precision-recall curve using the trapezoidal rule^44,47^, whereas the average precision is a weighted mean of precisions achieved at each threshold on the (non-interpolated) precision-recall curve, where the weights are set to be the increase in recall across the different thresholds^48^. We chose to report both the AUC-PR and the average precision since the trapezoidal rule used to estimate the AUC-PR can provide overly optimistic estimates for the precision-recall performance; both AUC-PR and average precision provide numerically close estimates for well-behaved precision-recall curves^49^. The *F*_1_ score is the harmonic mean of the model’s precision and recall; in Table 3 we compute each model’s *F*_1_ score at the cutoff point determined by its Youden’s J statistic.

AutoPrognosis outperformed the competing models with respect to all diagnostic metrics under consideration. We found the model developed by Nkam *et al*.^36^ to be the most competitive clinical model with respect to all metrics. All the results in Table 3 are statistically significant: 95% confidence intervals and *p*-values were obtained via 10-fold stratified cross-validation. All prognostic models performed markedly better than the simple criterion based on the FEV_1_ biomarker. AutoPrognosis displayed a satisfactory discriminative power, with an AUC-ROC of 0.89 (95% CI: 0.88–0.90) and a J statistic of 0.67 (95% CI: 0.65–0.69), outperforming the most competitive clinical model which achieves an AUC-ROC of 0.86 (95% CI: 0.85–0.87, *p*-value < 0.001) and a J statistic of 0.58 (95% CI: 0.55–0.61, *p*-value < 0.001). More importantly, AutoPrognosis displayed an even more significant gain with respect to the precision-recall performance metrics. In particular, it achieved an AUC-PR (Random guessing achieves an AUC-PR that is as low as 0.09.) of 0.58 (95% CI: 0.54–0.62), an average precision of 0.59 (95% CI: 0.55–0.63) and an *F*_1_ score of 0.60 (95% CI: 0.57–0.63), whereas the most competitive clinical model achieved an AUC-PR of 0.50 (95% CI: 0.47–0.53, *p*-value < 0.001), an average precision of 0.48 (95% CI: 0.45–0.51, *p*-value < 0.001) and an *F*_1_ score of 0.52 (95% CI: 0.50–0.54, *p*-value < 0.001).

We observe that the competing clinical models, albeit satisfying high AUC-ROC figures, are providing marginal (or no) gains with respect to the precision-recall metrics (The big gap between the AUC-PR and average precision values for the FEV_1_-based criterion reported in Table 3 resulted from the fact that this criterion creates a binary statistic with limited number of operating points, while the average precision is computed using the non-interpolated precision-recall curve.) For instance, the CF-ABLE-UK score achieves a better AUC-ROC compared to the FEV_1_-based criterion, but performs rather poorly in terms of the precision-recall measures since it additively combines the FEV_1_ predictors and many of the variables correlated with it, and hence it double-counts the risk factors for a large number of patients. (As we will show later, the CF-ABLE-UK score also ignores Oxygen therapy intake, which is an important variable for precise identification of low-FEV_1_ patients at risk.) The models developed by Nkam *et al*. and Buzzetti *et al*. achieve impressively high gains in AUC-ROC, but only modest gains in the AUC-PR and *F*_1_ scores, implying a limited clinical significance. Contrarily, AutoPrognosis was able to provide not only a high AUC-ROC figure, but also a significant improvement in the precision-recall metrics.

Finally, we checked whether the Bayesian optimization procedure used by AutoPrognosis was able to configure an ensemble of pipelines with tuned hyper-parameters that perform better than individual, plain vanilla machine learning benchmarks. We compared the diagnostic metrics of AutoPrognosis with those of 4 competitive machine learning algorithms: Gradient boosting, support vector machines (SVM), random forests and AdaBoost. As we can see in Table 3, the prognostic model learned by AutoPrognosis outperforms all the individual machine learning baselines, which highlights the benefit of using our framework instead of a naive deployment of off-the-shelf machine learning algorithms. We also compared the performance of AutoPrognosis with an open-source AutoML library known as *Tree-based Pipeline Optimization Tool*^50,51^ (TPOT), which uses a genetic algorithm to optimize and tune machine learning pipelines. The results in Table 3 show that AutoPrognosis clearly outperforms TPOT. In order to assess the benefits of Bayesian optimization and ensemble construction, we also evaluated the performance of the individual pipelines picked up by AutoPrognosis (Pipeline 1 and Pipeline 2 in Fig. 3) when tuned with grid and random search approaches. For a fixed computational cost (200 iterations), AutoPrognosis outperformed these benchmarks as well.

#### Assessing the clinical utility of AutoPrognosis

Practical deployment of a prognostic model in clinical decision-making would entail converting the model’s (continuous) outputs into binary decisions on whether a patient might be an appropriate candidate for transplant referral^10^. This can be achieved by setting a cutoff point on the model output (which corresponds to the patient’s risk), beyond which the patient is recommended for a transplant. In order to examine the potential impact of the prognostic models under study on clinical decision-making, we evaluated the diagnostic accuracy of AutoPrognosis, the best performing clinical model, and the FEV_1_-based criterion, at various cutoff points for transplant referral. The results are summarized in Table 4.

**Table 4:** Comparison of the diagnostic accuracy for the prognostic models under consideration at different cutoff points.

	Cutoff	PPV (95% CI) (%)	NPV (95% CI) (%)	Sens (95% CI) (%)	Spec (95% CI) (%)	Accuracy (%)	*F*_1_ score
FEV_1_% predicted	<20	66 (62, 70)	92 (91, 93)	13 (9, 17)	99 (98, 100)	92 (91, 93)	21 (19, 23)
	<30	48 (44, 52)	95 (94, 96)	46 (42, 50)	95 (94, 96)	91 (90, 92)	47 (45, 49)
	<40	29 (27, 31)	96 (95, 97)	62 (60, 64)	86 (84, 88)	84 (83, 85)	40 (38, 42)
	<50	21 (19, 23)	97 (96, 98)	73 (71, 75)	75 (73, 77)	75 (74, 76)	33 (31, 35)
Nkam *et al*.^36^	>6.5	75 (64, 86)	92 (91, 93)	13 (11, 15)	99 (98, 100)	92 (91, 93)	22 (19, 25)
	>4	56 (52, 60)	95 (94, 96)	46 (44, 48)	96 (95, 97)	92 (91, 93)	50 (49, 51)
	>2.5	42 (37, 47)	96 (95, 97)	61 (60, 62)	91 (90, 92)	88 (87, 89)	49 (45, 53)
	>2	31 (27, 35)	97 (96, 98)	73 (72, 74)	83 (79, 87)	82 (78, 86)	43 (39, 47)
	>0.50	88 (79, 97)	92 (91, 93)	13 (12, 14)	99 (98, 100)	92 (91, 93)	23 (22, 24)
AutoPrognosis	>0.33	65 (61, 69)	95 (94, 96)	46 (45, 47)	97 (96, 98)	93 (92, 94)	53 (51, 55)
	>0.15	49 (43, 55)	96 (95, 97)	62 (61, 63)	93 (92, 94)	90 (89, 91)	54 (50, 58)
	>0.10	36 (32, 40)	97 (96, 98)	74 (73, 75)	87 (86, 88)	86 (84, 88)	48 (45, 51)

In order to ensure a sensible comparison, sensitivity was fixed for all models at four levels (0.13, 0.46, 0.62, and 0.73); these are the four levels of sensitivity achieved by the FEV_1_ criterion at the cutoff thresholds 20%, 30%, 40% and 50%, respectively. The results in Table 4 show that at each cutoff threshold, the model learned via AutoPrognosis outperforms both the FEV_1_ criterion and the best performing competing model in terms of PPV, specificity, accuracy, and *F*_1_ scores. Of particular interest is the cutoff point of FEV_1_ < 30% (underlined in Table 4), which represents the main transplant referral criterion adopted in current clinical practices. The transplant referral policy achieving the same sensitivity as that achieved by the FEV_1_ < 30% criterion places a threshold of 0.33 on the output of AutoPrognosis. At this operating point, AutoPrognosis yields a PPV of 65%, which is significantly higher than that achieved by the FEV_1_ criterion (48%), and that achieved by the model developed by Nkam *et al*.^36^ (56%). That is, by adopting the model learned by AutoPrognosis for LT referral, we expect that the fraction of patients populating the lung transplant waiting list who are truly at risk would rise from 48% to 65%. In other words, in a waiting list of 100 patients, our model would replace 17 patients who were unnecessarily referred to a transplant with 17 other patients who truly needed one.

The clinical utility of AutoPrognosis is not limited to transplant referral; the predictions prompted by AutoPrognosis serve as granular risk scores that can quantify the severity of future outcomes and hence can be used for treatment planning, follow-up scheduling, or estimating the time at which a transplant would be needed in the future. For instance, decisions on whether a CF patient carrying a G551D mutation should start taking the (expensive) ivacaftor or lumacaftor drugs can be guided by the predictions of our model^52,53^. Patients with risk predictions that do not exceed the LT referral threshold are not equally healthy; higher risk scores are still indicative of higher levels of CF severity. The results in Tables 3 and 4 quantify the models’ ability to distinguish patients with and without poor (binary) outcomes (death or LT), but do not show how well the different models are able to predict less severe outcomes. To this end, we sought to classify the predictions of AutoPrognosis into low, moderate and high risk categories, and test the model’s ability to predict intermediate poor outcomes. We chose *pulmonary function decline* within a 3-year period as the intermediate poor outcome; we define pulmonary decline as the event when a patient has an FEV_1_% predicted less than 30% in the year 2015 (but did not undergo a lung transplant) when her FEV_1_% predicted was greater than 30% in 2012.

**Figure 5:**
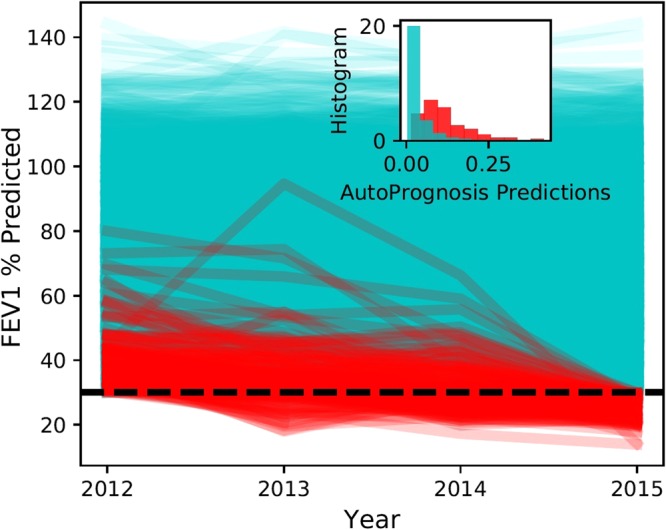
FEV_1_ trajectories over the years 2012 to 2015.

The FEV_1_ trajectories for all patients enrolled in the UK CF registry in 2012 are visualized in Fig. 5; FEV_1_ trajectories corresponding to pulmonary decline events are highlighted in red. The trajectories in Fig. 5 belong only to patients who had FEV_1_ > 30% in 2012 and did not die or undergo a transplant in 2015. A total of 4.4% of those patients experienced pulmonary function decline in 2015. The inset plot in Fig. 5 shows a histogram for the predictions of AutoPrognosis stratified by the occurrence of a pulmonary decline; we can visually see that AutoPrognosis is able to discriminate patients with and without the intermediate poor outcome. A two-sample *t*-test rejects the hypothesis that the average predictions for AutoPrognosis for patients with and without pulmonary decline are equal (*p*-value < 0.0001). The average predicted risk for patients without pulmonary decline was 0.046, whereas for those with pulmonary decline, the average predicted risk was 0.116. In order to assess the ability of our model to predict the pulmonary decline events, we redefined the poor outcomes as being death, lung transplant or pulmonary decline in a 3-year period. The in-sample average precision and AUC-PR of the predictive model learned by AutoPrognosis were 0.66 (95% CI: 0.63–0.69) and 0.65 (95% CI: 0.63–0.69), respectively, whereas those achieved by the model developed by Nkam *et al*. were 0.51 (95% CI: 0.48–0.54) and 0.48 (95% CI: 0.45–0.51). (95% confidence intervals were obtained via bootstrapping.) This demonstrates that AutoPrognosis is more precise than the existing models in predicting intermediate poor outcomes.

**Figure 6:**
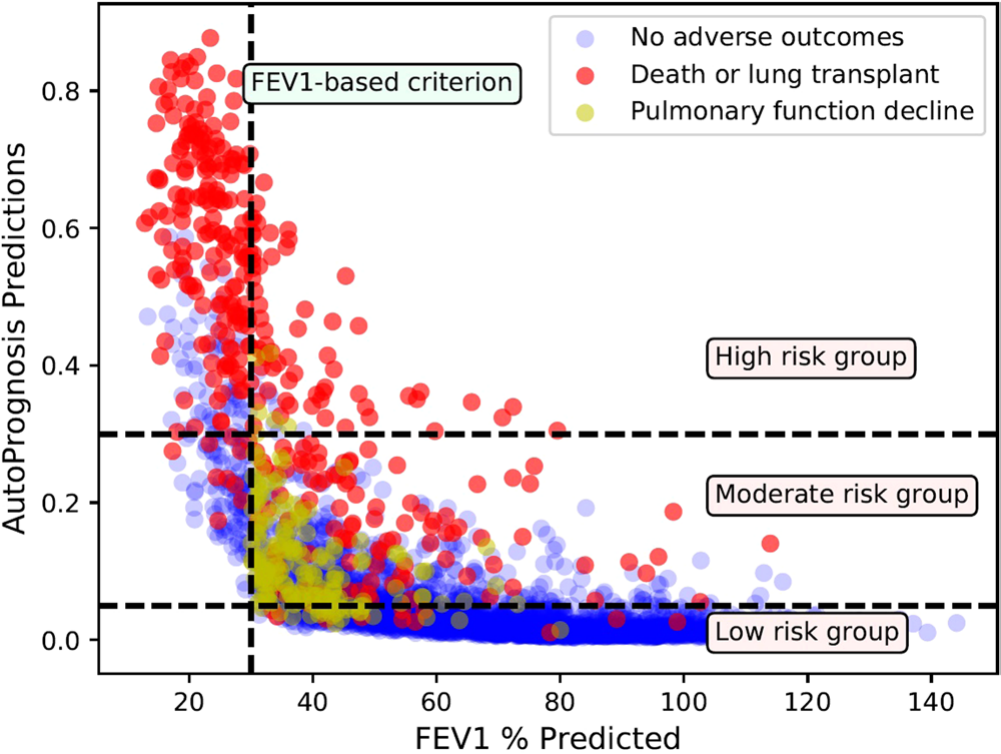
Depiction for the CF patients’ outcomes, FEV_1_ measures and predictions made by AutoPrognosis.

Predicated on the results above, we classified the CF population into three risk groups, with low, moderate and high risk, based on the risk predictions of AutoPrognosis. (In what follows, we converted the outputs of AutoPrognosis, which are real numbers between 0 and 1, into percentages.) The risk groups are defined as follows: the low risk group is associated with risk predictions in the range (0–5%), whereas the moderate risk group is associated with risk predictions in the range (5–30%), and finally, the high risk group is associated with risk predictions that exceed 30%. Figure 6 is a scatter plot for the CF patient outcomes in 2015 (red colored dots correspond to deaths or transplants, yellow dots correspond to pulmonary decline events, and blue dots correspond to patients with no adverse outcomes). The outcomes are plotted against the predictions issued by AutoPrognosis (*y*-axis), and every individual patient’s FEV_1_ measure in 2012 (*x*-axis). As we can see, the FEV_1_ criterion can only provide a low-precision classification of patients with and without the poor outcome, whereas AutoPrognosis provides a more precise risk stratification for the CF population in which most patients with intermediate poor outcomes (pulmonary decline) reside in the moderate risk group, and patient allocation to the high risk group exhibits lower false alarm rates (refer to Table 4). Clinicians can use the risk predictions and risk strata learned by AutoPrognosis as actionable information that guide clinical decisions. For instance, patients in the high risk group would be immediately referred to a transplant, patients in the moderate risk group would be recommended a drug with potential consideration for a transplant in the future, and patients in the low risk group should routinely pursue their next annual review.

### Variable importance

We sought to understand how the different patient variables contribute to the predictions issued by AutoPrognosis. Previous studies have identified a wide range of CF risk factors including FEV_1_% predicted^4,11,24,36,39^, female gender^4,24^, BMI^39,40^, Pseudomonas Aeruginosa infection^24^, Burkholderia cepacia colonization^36^, hospitalization^36^, CF-related diabetes^4,54^, non-invasive ventilation^36^, and ΔF508 homozygous mutation^24^. Since AutoPrognosis was trained in order to provide precise predictions, we focus not only on identifying variables that are most predictive of the outcomes in the sense of AUC-ROC maximization, but also on understanding which variables AutoPrognosis exploited in order to improve the precision (i.e. PPV) of the learned model (refer to Tables 3 and 4). These variables can then be considered when updating the current consensus guidelines on LT referral and waiting list priority allocation^12^.

**Figure 7:**
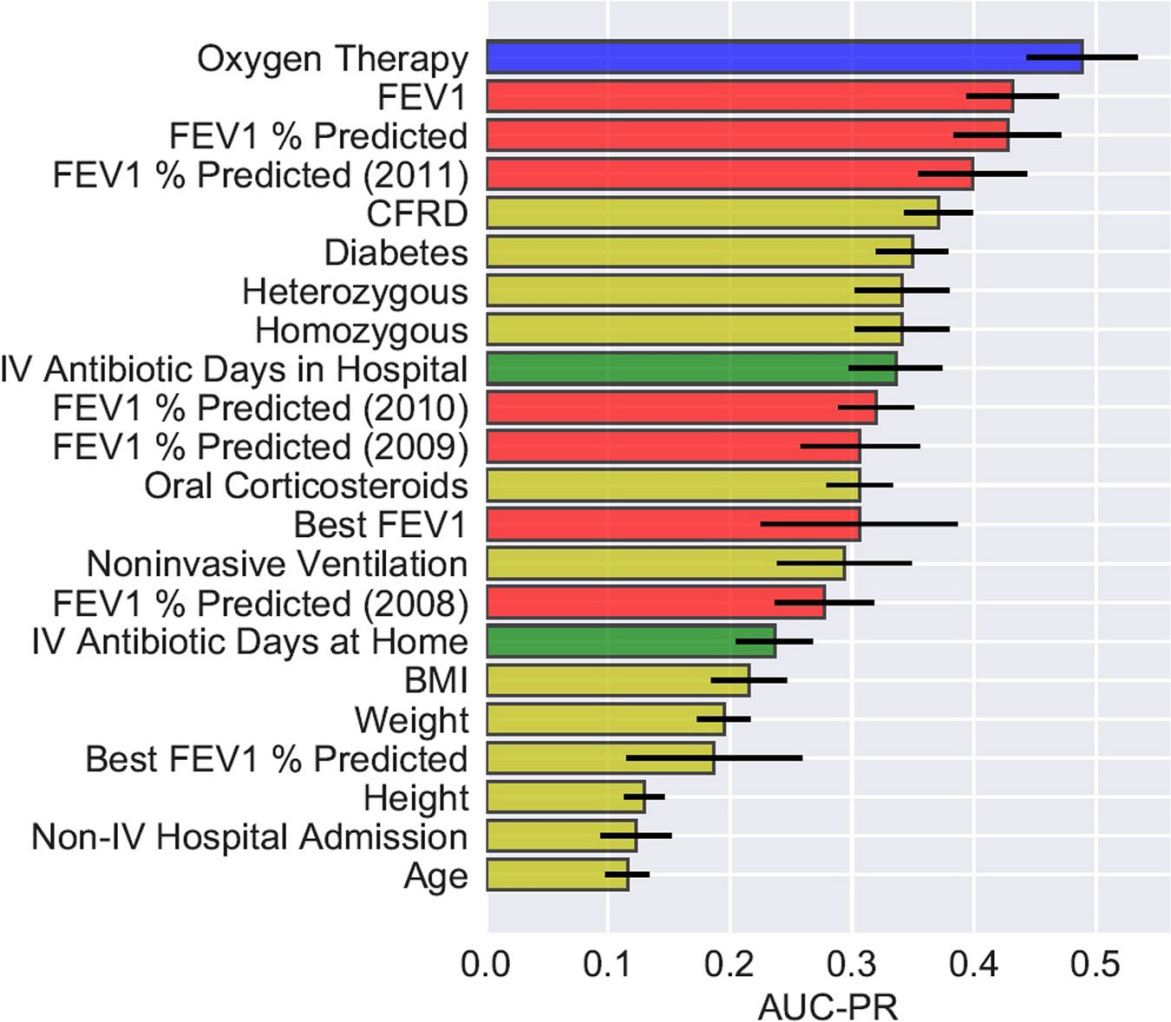
AUC-ROC of individual variables.

**Figure 8:**
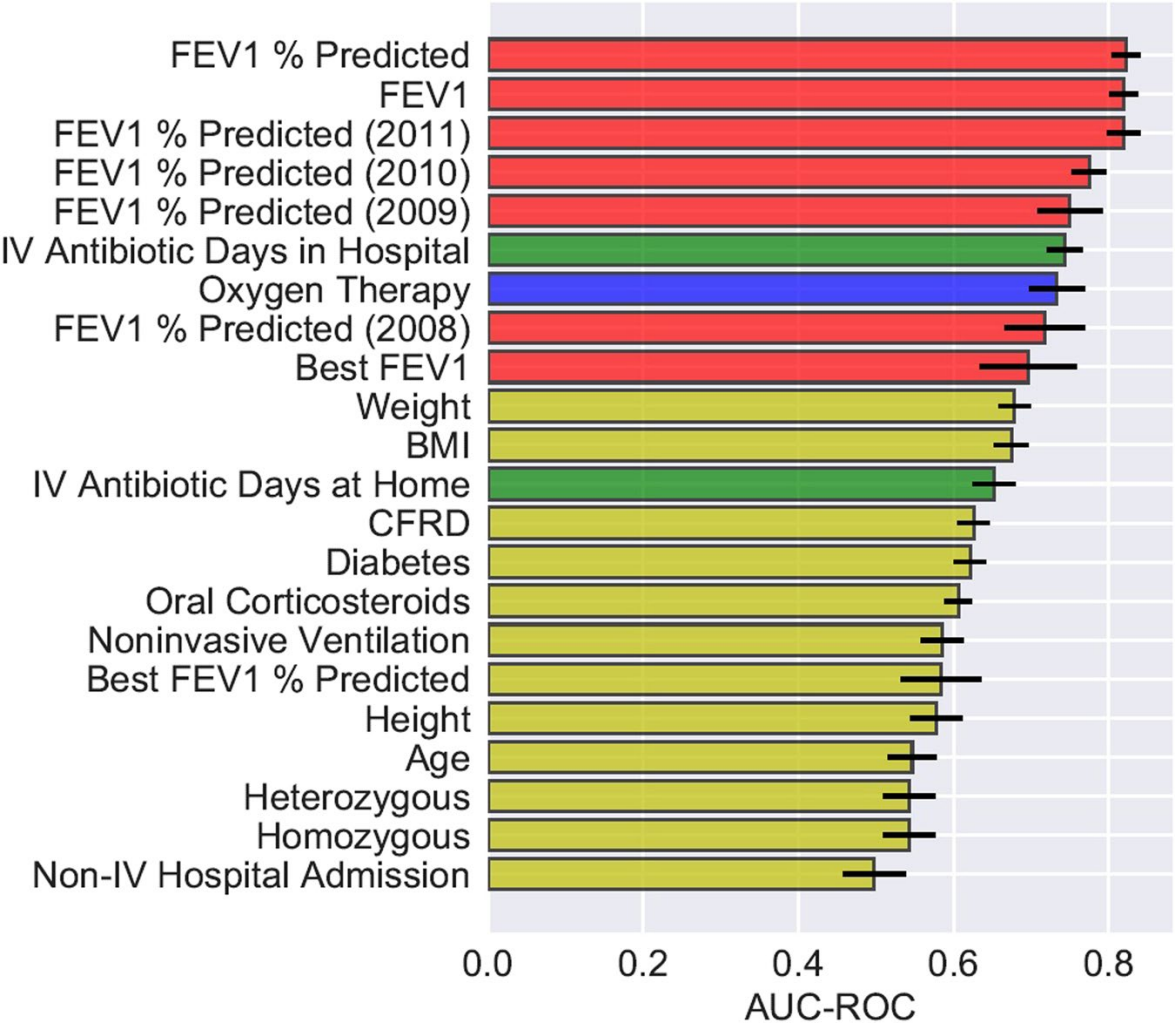
AUC-PR of individual variables.

We evaluated the predictive power of each individual variable by providing AutoPrognosis with one variable at a time, and assessing the diagnostic accuracy of the model that it constructs using only that variable. We evaluated the AUC-ROC and the AUC-PR metrics (using 10-fold stratified cross-validation) in order to get a full picture of each variable’s predictive power with respect to sensitivity, specificity, precision and recall. The most predictive 22 variables with respect to both the AUC-ROC and the AUC-PR metrics are illustrated in Figs 7 and 8. In both figures, the bars associated with the variables correspond to the AUC-ROC/AUC-PR performance achieved by AutoPrognosis using only this variable. The black error bars correspond to the 95% confidence intervals. Since CF patients may encounter pulmonary disorders manifesting in either increased *airway resistance* or impaired *gas exchange*^55^, we labeled the patients’ variables in Figs 7 and 8 based on the aspect of lung function that they reflect. Variables that describe lung function in terms of airway resistance (e.g. FEV_1_, FEV_1_% predicted, FEV_1_ trajectory, etc) are represented through red bars. Variables that describe lung function in terms of gas exchange (e.g. Oxygenation) are represented through blue bars. Variables that represent pulmonary disorders resulting from bacterial infections are represented through green bars. All other variables had their corresponding bars colored in yellow.

Figure 7 shows that the spirometric (FEV_1_) biomarkers, including the FEV_1_ measurements collected 3 years prior to 2012, display the best AUC-ROC performance. Interestingly, we found that the history of FEV_1_ measurements (e.g. the FEV_1_% predicted 1 year before baseline) is as predictive as the FEV_1_ measurements at baseline. Variables reflecting pulmonary disorders resulting from bacterial infections (intravenous antibiotic courses in hospital^56^) were the second most predictive in terms of the AUC-ROC performance. The most predictive complications were found to be diabetes and CF-related diabetes. Apart from intravenous antibiotics, the most predictive treatment-related variable was usage of oral corticosteroids. Genetic variables and microbiological infections were found to have a poor predictive power when used solely for predictions, though intravenous antibiotic courses can be thought of as proxies for microbiological infections.

Figure 8 shows that the importance ranking for the patients’ variables changes significantly when using precision (i.e. AUC-PR) as a measure of the variables’ predictive power. Most remarkably, reception of Oxygen therapy turns out to be the variable with the highest AUC-PR. Hence, precise risk assessment and transplant referral decisions need to consider, in addition to the spirometric biomarkers, other biomarkers that reflect disorders in gas exchange, such as the partial pressure of carbon dioxide in arterial blood (PaCO_2_) and Oxygen saturation by pulse oximetry (SpO_2_)^57^. Prevalence of respiratory failures that are usually treated via Oxygenation, such as hypoxemia and hypercapnia^17,55,57,58^, should be considered as decisive criteria for LT referral even when airway obstruction is not severe (i.e. FEV_1_ > 30%). AutoPrognosis was able to learn a prediction rule that carefully combines spirometric and gas exchange variables in order to come up with a precise lung transplant referral criterion that accurately disentangles patients who are truly at risk from those who do not need a lung in the near future (refer to Tables 3 and 4). Our results indicate that looking at the right accuracy metric that reflects the true clinical utility (in this case the precision-recall curve) is important not only for tuning and comparing predictive models, but also for discovering risk factors that are relevant for clinical decision-making.

**Figure 9:**
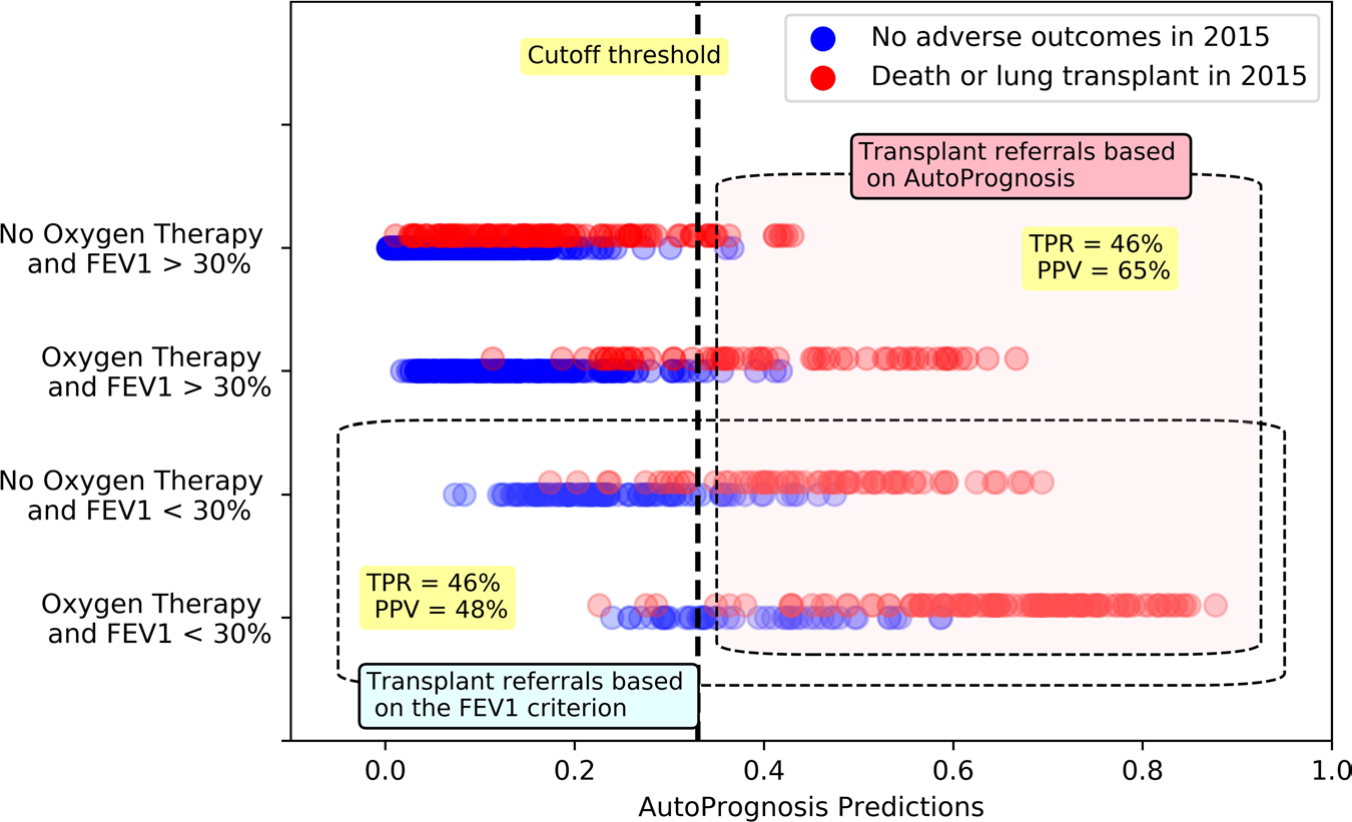
Depiction for transplant referral policies based on AutoPrognosis and the FEV_1_ criterion for different patient subgroups.

**Figure 10:**
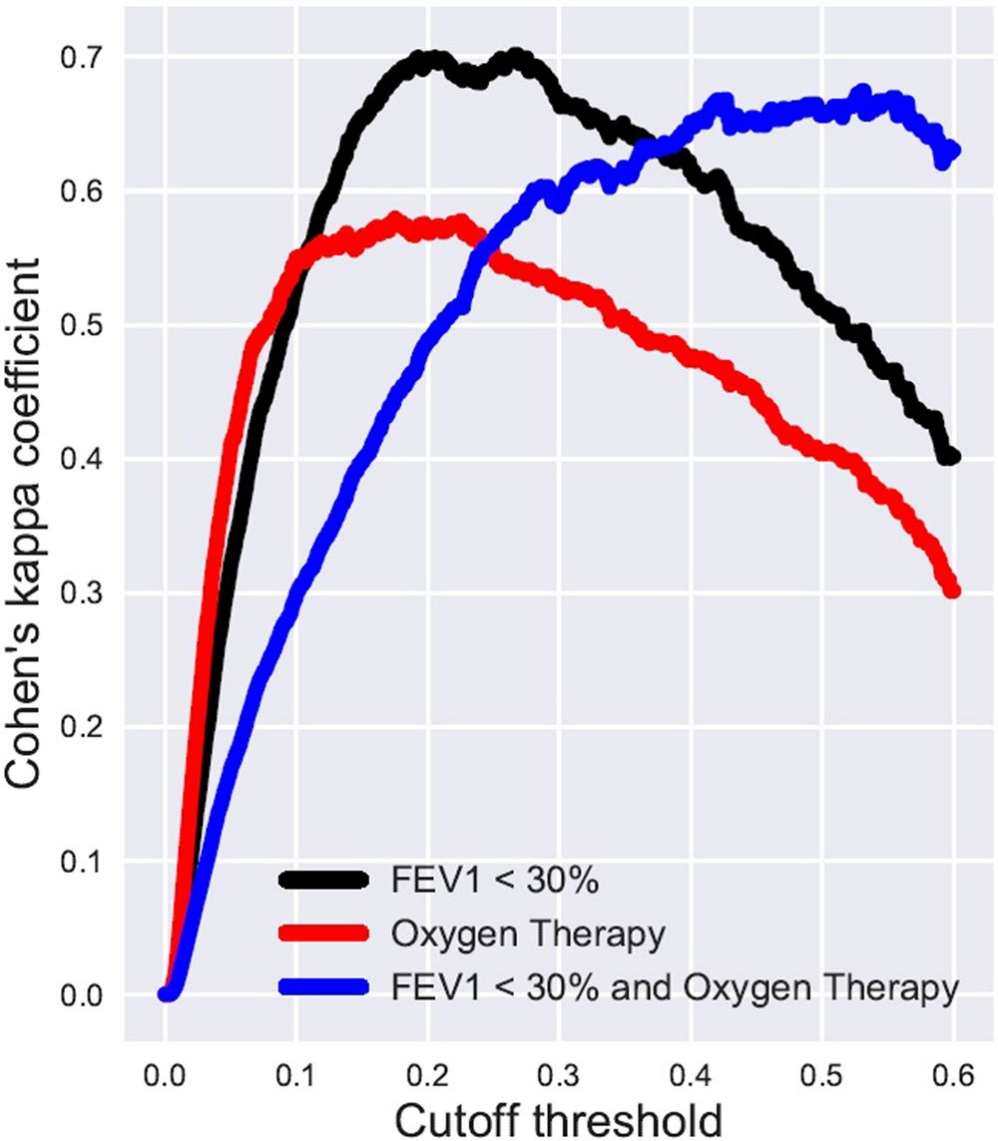
Inter-rater agreement between AutoPrognosis and 3 simple referral policies.

Figures 9 and 10 illustrate how LT referral policies based on AutoPrognosis handle patient subgroups stratified by spirometric and Oxygenation variables. In Fig. 9, we look at 4 subgroups: patients with FEV_1_ < 30% who received Oxygen therapy, patients with FEV_1_ < 30% who did not receive Oxygen therapy, patients receiving Oxygen therapy but had FEV_1_ ≥ 30%, and patients who were neither Oxygenated nor had their FEV_1_ drop below the 30% threshold. The subgroup memberships are labeled on the *y*-axis; every patient is represented as a dot in a scatter plot, with the *x*-axis quantifying the risk estimate of AutoPrognosis for every individual patient. Patients with adverse outcomes are represented via red dots, whereas those with no adverse outcomes are depicted as blue dots. As we can see in Fig. 9, the simple FEV_1_ criterion would refer the two subgroups with poor spirometric biomarkers (FEV_1_ < 30%) to a transplant; this leads to a referral list with many blue dots (this is depicted via the dotted box that groups all patients with FEV_1_ < 30% in Fig. 9), and consequently a high false positive rate that leads to a PPV of 48%. Contrarily, AutoPrognosis orders the risks of the 4 subgroups by accounting for both Oxygenation and spirometry; this results in a more precise list of referrals at any given cutoff threshold (as can be seen in the dotted box that groups all patients with risk cutoff of 0.33, where the majority of the dots in the box are red). AutoPrognosis achieves precision by assigning a high risk assessment to Oxygenated patients, even if their spirometric biomarkers are not severe. At a fixed TPR of 46%, this leads to some of the patients with FEV_1_ < 30% but good clinical outcomes being replaced with Oxygenated patients with FEV_1_ > 30% who experienced adverse outcomes, which raises the PPV to 65%. Figure 10 illustrates the agreement between a lung transplant referral policy based on AutoPrognosis and 3 policies that make referral decisions based on either spirometry, Oxygenation or both. As we can see, the higher the cutoff threshold is (high cutoff threshold means that the length of the waiting list is restricted, perhaps due to scarcity of donors), the more similar is the policy based on AutoPrognosis to a policy that picks patients who were both Oxygenated and had an FEV_1_ below 30%. This implies that AutoPrognosis ranks the patients’ risks by incorporating both spirometric and gas exchange variables, and hence in a practical setting in which patients are already in a transplant waiting list, AutoPrognosis would assign higher priorities to patients who encountered problems with both airway obstruction and impaired gas exchange.

### AutoPrognosis’ Interpreter

The variable importance analyses conducted (manually) in the previous subsections aimed at “interpreting” the prognostic model learned by AutoPrognosis, and revealed interesting insights on the role of different variables in clinical decision-making. As a part of our automated framework, we sought to automate the process of interpreting the complex prognostic model learned by AutoPrognosis and distilling clinical insights from its underlying prediction rule. That is, we want AutoPrognosis to automatically “explain” its own predictions. Since models that are inherently interpretable, such as decision trees, often exhibit modest predictive accuracy^33^, we chose to separate the problem of tuning the predictive model from the problem of explaining its predictions^34^. This was achieved by supplying AutoPrognosis with a post-processing module, which we call the “interpreter” (see Fig. 1), which operates on the (arbitrarily complex) prognostic model generated by the preceding Bayesian optimization module, and attempts to extract association rules that link different actionable variables to risk strata that are predefined by clinicians. It is important to note that the interpreter’s role is only to *explain* the predictions of the prognostic model and is not used for issuing any *predictions*, and hence we do not construct the interpreter to optimize any accuracy metric.

**Figure 11:**
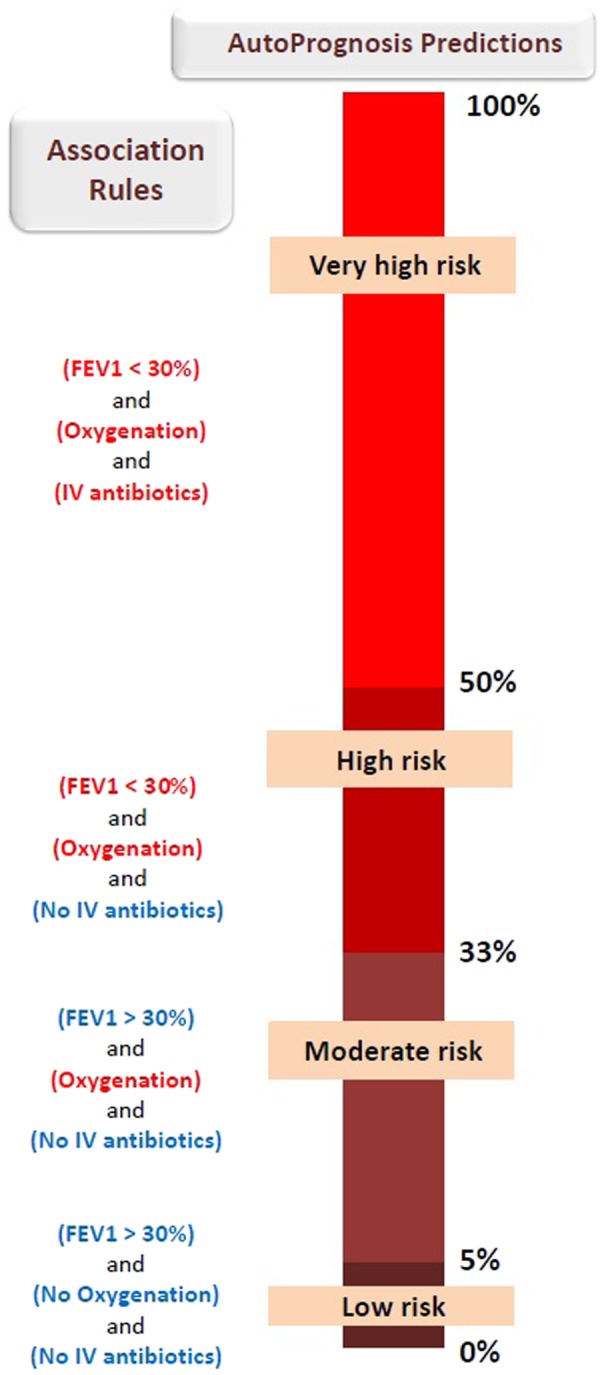
The interpreter’s association rules.

The interpreter module is implemented as a simple associative classifier^59^ which can be expressed through a set of clinically interpretable association rules (*if-then rules*) that link conjunctions of clinical conditions to risk predictions. An example for a possible association rule is: *if* the patient had her FEV_1_ below 30% *and* had a B. Cepacia infection, *then* the patient will need a LT within the next 3 years. Implementation details for the associative classifier used in the interpreter module are provided in Methods. Figure 11 depicts the statistically significant association rules discovered by AutoPrognosis’ interpreter for a predefined risk strata that clusters the patients into 4 risk groups, where we can see that the interpreter managed to automatically reconstruct the insightful findings presented in the previous subsections.

### Sensitivity analyses

AutoPrognosis was able to identify Oxygenation as a key variable for ensuring precise predictions for CF patients. We tested the robustness of this finding by examining the impact of defining a composite endpoint of death and transplant on our analysis of variable importance. In particular, we conducted a *Chi-Square test* of independence in order to test whether an association between Oxygenation and transplant events (rather than death events) existed in the data and led to the importance ranking in Fig. 8. (The test was conducted on the population of patients with poor outcomes.) With a *p*-value of 0.723, the hypothesis of Oxygenation being associated with transplants through an underlying clinical policy was rejected. The variable importance ranking in Fig. 8 did not change when defining importance via F_1_ scores (see Supplementary Table S1).

Patients lost to follow-up are unlikely to have had an impact on our findings. By imputing the outcomes of patients lost to follow-up, we found their mean risk (as predicted by AutoPrognosis) to be 7.20% ± 0.29%, as compared to a mean risk of 8.54% ± 0.45% for the study population. A two-sample *t*-test rejected the null hypothesis that the two populations have the same average risk (*p*-value < 0.001), and suggested that the patients lost to follow-up had a lower average risk. In order to examine the impact of not including those patients in our study, we augmented all patients lost to follow-up to our study population, and constructed an “adversarial” dataset in which none of the patients lost to follow-up had experienced a poor outcome. Such a dataset is “adversarial” to our findings since assuming that all patients lost to follow-up were alive implies that those patients had a distribution for their outcomes that does not match the observed event incidences used to tune our model, and is instead set to an extreme distribution that could change our conclusions. By feeding the augmented data set to AutoPrognosis, the variable importance rankings in Figs 7 and 8 did not change (see Supplementary Table S2). In the supplementary material, we also provide a table showing the variable importance ranking when correlations between features are accounted for using the correlation-based feature selection (CFS) method^60^ (see Supplementary Table S3). The CFS algorithm assigned high ranks to both the FEV1 and Oxygen therapy variables. However, since CFS is based merely on feature-class (and feature-feature) correlations, it was not possible for the CFS-based ranking to disentangle the differences between FEV_1_ and Oxygen therapy variables in terms of accuracy and precision. Moreover, since CFS accounts for correlations among features, it discarded some of the FEV_1_ measures that were deemed important in the single-variable analysis as they were correlated with the patient’s most recent FEV_1_ % predicted measure.

## Discussion

In this paper, we developed an algorithmic framework that leverage Bayesian optimization techniques for automating the process of constructing machine learning-based clinical prognostic models. Our framework allows clinical researchers to efficiently build highly-optimized machine learning pipelines for prognostication without the need for complicated design choices or time-consuming manual tuning of models’ hyper-parameters, which require in-depth technical expertise. Our framework also allows for interpreting complex machine learning models by mining for association rules that relate conjunctions of clinical conditions with risk strata.

We applied our general framework to the problem of predicting short-term survival of cystic fibrosis patients using data from the UK CF registry. AutoPrognosis was capable of learning an ensemble of machine learning models (including the well-known random forest and XGBoost algorithms) that outperformed existing risk scores developed in the clinical literature, mainstream practice guidelines, and naïve implementation of vanilla machine learning models. We demonstrated the clinical utility of the prognostic model learned by AutoPrognosis by examining its potential impact on lung transplant referral decisions. Our analysis showed that the model learned by AutoPrognosis achieves significant gains in terms of a wide variety of diagnostic accuracy metrics. Most notably, AutoPrognosis achieves significant gains in terms of the positive predictive values, which implies a remarkable improvement in terms of the precision of lung transplant referral decisions. AutoPrognosis’ interpreter module revealed that the model is able to achieve such gains because it recognizes the importance of variables that reflect disorders in pulmonary gas exchange (such as Oxygenation), and learns their interactions with spirometric biomarkers reflecting airway obstruction (such as FEV_1_). This gave rise to a precise survival prediction rule which disentangles patients who are truly at risk from those who do not necessarily need a transplant in the short term.

Although our study provided empirical evidence for the clinical usefulness of applying automated machine learning in prognostication, it has some limitations. First, the prognostic model learned by AutoPrognosis needs to be externally validated in order to ensure that our findings generalize to other CF populations. Second, the net clinical utility of our model needs to be evaluated by considering post-transplant survival data, through which we can identify high-risk patients for whom a transplant is indeed beneficial. Finally, we had no access for data on patients who went through a transplant evaluation process or were enrolled in wait list but did not get a transplant within the 3-year analysis horizon, which rendered direct comparisons with the actually realized clinical policy impossible.

## Methods

### Data and clinical prognostic models

The UK CF registry comprises annual follow-up data for a total of 10,980 CF patients over a period spanning between 2008 and 2015. Data was gathered at every specialist center and clinic across the UK, with 99% of patients consenting to their data being submitted^26^, and hence the cohort is representative of the UK CF population. Variables with highest rates of missingness were those related to the spirometric trajectory: the missingness rates for the patients’ FEV_1_ biomarkers in 2008, 2009, 2010, 2011 and 2012 were 31.0%, 20.0%, 15.5%, 6.2% and 4.4%, respectively. The missingness rates in the height, weight and BMI variables were 2.1%, 1.4% and 2.9%, respectively. Since AutoPrognosis software picked the missForest imputation algorithm in all cross-validation folds, an imputed dataset was created using the missForest algorithm for all the competing methods to ensure a fair comparison. Mortality data were extracted from the perennial database maintained by the UK CF trust, which includes all the death events for CF patients up to December 31^*st*^, 2015, including death events for patients who did not provide annual review data in 2012. We did not assume that patients who neither have shown up for the annual review in 2015 nor had been included in the death records in the perennial database to be alive. Instead, we assumed that those patients were lost to follow-up; the sensitivity analysis conducted earlier shows that our results would not change had we assumed those patients to be alive by 2015. We excluded from our analysis all patients who have had a LT at anytime that preceded her 2012 annual follow-up. We had no access to LT data in the years before 2008, but it is unlikely that this would affect our results since the number of LT that were carried out before 2008 is negligible compared to the size of the study population.

We were not able to implement the CF-ABLE score by McCarthy *et al*.^39^, which uses the number of pulmonary exacerbations as one of its three risk predictors, as the UK CF registry does not keep track of exacerbations. Instead, we implemented the modified CF-ABLE-UK score^40^, which uses the number of days the patient required intravenous antibiotics as a proxy for exacerbations. We were able to replicate the validation results obtained previously by Dimitrov *et al*.^40^; the in-sample AUC-ROC of CF-ABLE-UK in our study population was 0.7997, which closely matches the AUC-ROC of 0.80 (95% CI: 0.79–0.83) reported in their study. The models developed by Nkam *et al*.^36^ and Buzzetti *et al*.^23^ were fit to the French and Italian CF populations, respectively. Both models were re-calibrated prior to the diagnostic accuracy evaluations conducted in the Results section: the model by Nkam *et al*. was recalibrated in the large to match the incidence of poor outcomes in the UK CF population, whereas the model by Buzzetti *et al*. had its coefficients adjusted using logistic recalibration.

### Implementation of AutoPrognosis

AutoPrognosis is implemented as an installable Python package, with some of its submodules implemented in R and interfaced with the Python module via RPy2-based wrappers. Bayesian optimization was implemented using GPyOpt^61^; a Python library that is based on GPy^62^. Currently, AutoPrognosis supports 7 imputation algorithms, 14 feature processing algorithms, 20 classification algorithms, and 3 calibration methods. Thus, AutoPrognosis can build prognostic models that combine any subset of a total of 5,460 machine learning pipelines. The 7 imputation algorithms are: mean imputation, median imputation, most-frequent imputation, expectation-maximization (EM), matrix completion, multiple imputation by chained equations (MICE), and missForest. Through RPy2-based wrappers, AutoPrognosis uses the R libraries mice, Amelia, softImpute and missForest to implement the MICE, EM, matrix completion and missForest imputation algorithms, respectively. The calibration methods considered by AutoPrognosis are: sigmoid regression, isotonic regression, or no calibration. The feature processing and classification algorithms deployed in the AutoPrognosis framework include all elements of the Scikit-learn Python library^63^. For feature processing, this includes (but not limited to) PCA, kernel PCA, feature agglomeration, fast ICA, random kitchen sinks, linear SVM preprocessing, Nystroem sampler, polynomial feature processing, and random trees embeddings. For classification algorithms, AutoPrognosis includes Gradient boosting, XGBoost, random forest, Naive Bayes, AdaBoost, Bagging, linear and kernel SVM, etc.

#### Bayesian optimization and ensemble construction

AutoPrognosis uses a Bayesian optimization approach to configure and combine machine learning pipelines with the goal of optimizing a given clinical utility function. In what follows, we present the formulation and algorithmic details of the AutoPrognosis training procedure. Let ${\mathscr{D}}={({X}_{i},{y}_{i})}_{i=1}^{n}$ be the training dataset, where *X*_*i*_ is the variables of patient *i* in 2012, and ${y}_{i}\in \{0,1\}$ is a binary label that is set to 1 if the patient encountered an adverse outcome by 2015. Let ${\mathscr{P}}$ denote the set of all pipelines supported by AutoPrognosis. Each pipeline $P(\theta )\in {\mathscr{P}}$ has a set of hyper-parameters that belongs to some hyper-parameter space Θ, i.e. $\theta \in {\rm{\Theta }}$. Let $U(P(\theta ),{\mathscr{D}})$ be an empirical estimate of the clinical utility achieved with pipeline *P* and hyper-parameter *θ*. Thus, AutoPrognosis attempts to solve the following optimization problem

1 $${P}^{\ast },{\theta }^{\ast }=\mathop{{\rm{\arg }}\,{\rm{\max }}}\limits_{P(\theta )\in {\mathscr{P}},\theta \in {\rm{\Theta }}}\,U(P(\theta ),{\mathscr{D}}).$$

Since we have no closed-form expression or gradient information for the complex objective function in Equation 1, we follow a “black box” optimization approach in which we repeatedly query the objective $U(P(\theta ),{\mathscr{D}})$ for different selections of the pipelines *P* and hyper-parameters *θ*. Note that every pipeline *P* can be decomposed into a set of “stages”, i.e. *P* = {*I*(*θ*), *F*(*θ*), *M*(*θ*), *C*}, where *I* is the imputation stage, *F* is the feature processing stage, *M* is the classification stage and *C* is the calibration stage. Note that the 3 calibration algorithms utilized by AutoPrognosis have no hyper-parameters to be tuned. In order to simplify the optimization problem, we decouple the imputation and calibration stages from the other stages of the pipeline, i.e. we optimize the following approximate clinical utility:

2 $${P}^{\ast },{\theta }^{\ast }=\mathop{{\rm{\arg }}\,{\rm{\max }}}\limits_{P(\theta )\in {\mathscr{P}},\theta \in {\rm{\Theta }}}\,{\tilde{U}}_{c}(C,{\mathscr{D}})+\tilde{U}(M({\theta }_{M}),F({\theta }_{F}),{\mathscr{D}})+{\tilde{U}}_{I}(I({\theta }_{I}),{\mathscr{D}}),$$

where ${\tilde{U}}_{c}(C,{\mathscr{D}})$ is the clinical utility achieved by the calibration algorithm *C*, $\tilde{U}(M({\theta }_{M}),F({\theta }_{F}),{\mathscr{D}})$ is the utility achieved by a combination of a feature processing *F* and classification algorithm *M* with hyper-parameters *θ*_*F*_ and *θ*_*M*_, and ${\tilde{U}}_{I}(I({\theta }_{I}),{\mathscr{D}})$ is utility achieved by the imputation algorithm *I* with hyper-parameters *θ*_*I*_. The approximation in Equation 2 assumes that the performance of calibration and imputations algorithms does not depend on the feature processing and classification algorithms. Hence, the optimization problem in Equation 2 can be decoupled into 3 separate optimization problems as follows:

3 $$\begin{array}{rcl}{M}^{\ast },{\theta }_{M}^{\ast },{F}^{\ast },{\theta }_{F}^{\ast } & = & \mathop{{\rm{\arg }}\,{\rm{\max }}}\limits_{M,{\theta }_{M},F,{\theta }_{F}}\,\tilde{U}(M({\theta }_{M}),F({\theta }_{F}),{\mathscr{D}}),\\ {I}^{\ast },{\theta }_{I}^{\ast } & = & \mathop{{\rm{\arg }}\,{\rm{\max }}}\limits_{I,{\theta }_{I}}\,{\tilde{U}}_{I}(I({\theta }_{I}),{\mathscr{D}}),\\ {C}^{\ast } & = & \mathop{{\rm{\arg }}\,{\rm{\max }}}\limits_{C}\,{\tilde{U}}_{c}(C,{\mathscr{D}}).\end{array}$$

Let ${\mathcal M}$, ${\mathcal F}$, and ${\mathscr{J}}$ be the spaces of all possible classification, feature processing and imputation algorithms and their corresponding hyper-parameters. AutoPrognosis follows a Bayesian optimization approach for solving the 3 optimization problems in Equation 3, where we place a Gaussian process prior over the clinical utility functions as follows^32^

4 $$\begin{array}{rcl}\tilde{U}(M({\theta }_{M}),F({\theta }_{F}),{\mathscr{D}}) & \sim  & GP(0,{{\bf{K}}}_{M}),\\ {\tilde{U}}_{I}(I({\theta }_{I}),{\mathscr{D}}) & \sim  & GP(0,{{\bf{K}}}_{I}),\\ {\tilde{U}}_{c}(C,{\mathscr{D}}) & \sim  & GP(0,{{\bf{K}}}_{C}),\end{array}$$

where *GP*(0, **K**_*M*_), *GP*(0, **K**_*I*_) and *GP*(0, **K**_*C*_) are Gaussian process priors (with kernels **K**_*M*_, **K**_*I*_ and **K**_*C*_) defined over the input spaces ${\mathcal M}$, ${\mathcal F}$, and ${\mathscr{J}}$, respectively. We chose the adaptive Matern 3/5 kernel for all the kernel functions **K**_*M*_, **K**_*I*_ and **K**_*C*_. The Gaussian process priors allows AutoPrognosis to easily compute posterior beliefs about the clinical utility of all possible pipelines in closed-form. AutoPrognosis uses an *acquisition function* derived from the Gaussian process posterior in order to guide a sequence of evaluations of the clinical utility functions $\tilde{U}$, ${\tilde{U}}_{I}$ and ${\tilde{U}}_{c}$ in order to figure out the best pipeline. The acquisition function is designed so as to help AutoPrognosis balance between exploring new pipelines and re-evaluating previously explored ones. We use an *Upper Confidence Bound* acquisition function, which at the *K*^*th*^ iteration of the sequential algorithm is given by:

5 $$\begin{array}{rcl}a((M,F);{\{({M}_{k},{F}_{k})\}}_{k=1}^{K-1}) & = & \mu ((M,F);{({M}_{k},{F}_{k})}_{k=1}^{K-1})-\kappa \sigma ((M,F);{({M}_{k},{F}_{k})}_{k=1}^{K-1}),\\ a(I;{\{{I}_{k}\}}_{k=1}^{K-1}) & = & \mu (I;{\{{I}_{k}\}}_{k=1}^{K-1})-\kappa \sigma (I;{\{{I}_{k}\}}_{k=1}^{K-1}),\\ a(C;{\{{C}_{k}\}}_{k=1}^{K-1}) & = & \mu (C;{\{{C}_{k}\}}_{k=1}^{K-1})-\kappa \sigma (C;{\{{C}_{k}\}}_{k=1}^{K-1}),\end{array}$$

where *μ* and *σ* are the posterior means and variances of the 3 Gaussian processes, and a *κ* is a tunable parameter that balances exploration and exploitation. We dropped the notations for hyper-parameters in Equation 5 for the sake of brevity. The sequential exploration and exploitation procedure goes as follows:

**In the** ***K*** ^***th***^ **step:**

1. Select the calibration algorithm *C*_*K*_, feature processing algorithm *F*_*K*_, imputation algorithm *I*_*K*_ and classification algorithm *M*_*K*_ so as to maximize the acquisition functions $a((M,F);{\{({M}_{k},{F}_{k})\}}_{k=1}^{K-1})$, $a(I;{\{{I}_{k}\}}_{k=1}^{K-1})$ and $a(C;{\{{C}_{k}\}}_{k=1}^{K-1})$.
2. Evaluate the clinical utilities $\tilde{U}$, ${\tilde{U}}_{I}$ and ${\tilde{U}}_{c}$ using cross-validation.
3. Update the posterior means and variances *μ* and *σ*.
4. Update the acquisition functions and repeat step 1.

After convergence, AutoPrognosis constructs an ensemble of pipelines, which we call a *super-pipeline*, by assigning every pipeline with a weight that is equal to the probability that the pipeline has the highest clinical utility among all the ones that have been evaluated, i.e. the weight of pipeline *P* is given by ${\mathbb{P}}({\{U(P;{\mathscr{D}}) > U({P}_{k};{\mathscr{D}})\}}_{k})$, where {*P*_*k*_}_*k*_ is the set of all the evaluated pipelines. The probability ${\mathbb{P}}({\{U(P;{\mathscr{D}}) > U({P}_{k};{\mathscr{D}})\}}_{k})$ can be easily evaluated by virtue of the conjugacy of the Gaussian process posterior. We defined the clinical utility as the average of the area under precision-recall curve and the average precision metrics in order to maximize the model’s positive predictive values.

#### The interpreter

The interpreter module is a post-processing algorithm that takes as an input the optimized super-pipeline (P*) found by the Bayesian optimization module, and risk strata decided by clinicians. The risk strata is defined as a set ${\mathcal R}$ comprising *M* intervals that partition the $[0,\,1]$ interval, and represent distinct sets of actionable risk groups for which different clinical decisions would be made, i.e.

$$ {\mathcal R} =\{[0,{r}_{1}),[{r}_{1},{r}_{2}),\ldots ,[{r}_{M-1},{r}_{M}]\},\,{r}_{k}\in [0,1],\,{r}_{k} > {r}_{j},\forall k,\,j\in \{1,\ldots ,M\},\,k > j.$$

A potential risk stratification for the CF population is the one given in Fig. 11, which can be represented by the set ${\mathcal R} =\{[0,0.05),[0.05,0.33),[0.33,0.5),[0.5,1]\}$. This strata divides the CF population into low risk, moderate risk, high risk and very high risk groups. The corresponding actions could be: continue annual follow-ups, administer a drug (e.g. inhaled antibiotic), refer to a LT, refer to a LT with a high priority allocation in the waiting list. The interpreter’s objective is to interpret the complex risk scoring function embedded in the super-pipeline P* through easy-to-understand logical associations between clinical conditions and the predefined risk strata. The outputs of the interpreter are of the form:

$${C}_{1}\wedge {C}_{2}\wedge \ldots \wedge {C}_{l(R)}\Rightarrow R,\,\forall R\in  {\mathcal R} ,$$

where {*C*_1_, *C*_2_, …, *C*_*l*(*R*)_} is a set of the *l*(*R*) boolean conditions associated with risk group *R*. An example for an association discovered by the interpreter, which is depicted in Fig. 11, is

$$({{\rm{FEV}}}_{1} < 30 \% )\wedge ({\rm{No}}\,{\rm{Oxygenation}})\wedge ({\rm{No}}\,{\rm{IV}}\,{\rm{antibiotics}})\Rightarrow {\rm{Low}}\,{\rm{risk}},$$

where for this risk group we have that *l*(Low risk) = 3, i.e. three clinical conditions are associated with membership in the low risk group. The association rules are aimed at explaining the reason why AutoPrognosis makes certain predictions, which conjunctions of medical conditions lead to higher risk predictions, and which variables are more important for assessing a patient’s short-term risk for adverse outcomes. These explanations are not only useful for debugging the clinical sensibility of the knowledge that AutoPrognosis has acquired from the data, but it can also help clinicians make decisions by presenting them with a simple rules that map conditions to outcomes. In other words, the interpreter tries to present the clinicians with a “data-driven practice guideline”.

The interpreter mines for association rules through the following three-step *associative classification* procedure^59,64^:

- **Step 1:** Discretize continuous variables.
- **Step 2:** Mine for all class association rules.
- **Step 3:** Prune the discovered association rules using minimum support and minimum confidence constraints.

Step 1 involves discretizing the continuous variables using the minimum description length principle^65^. AutoPrognosis uses the CBA (Classification Based on Associations) classifier to implement the three steps above^64^, with the exception of the FEV_1_ biomarker which is discretized using the 30% threshold. The discretization process conducted by AutoPrognosis allows the user to either let the variables be discretized automatically or manually of clinicians are interested in particular ranges of the given biomarkers. Through the CBA classifier, the class association rules are identified as follows. After discretizing the continuous variables in Step 1, we have a relational dataset with categorical attributes and risk strata as classification targets. Every possible realization of a categorical variable and risk category corresponds to an association rule, or a *ruleitem*. An association rule holds with confidence *c*% and support *s*% if it holds for *c*% of the patients in the dataset, and the corresponding risk group has a prevalence of *s*%. We use the greedy *k*-ruleitem algorithm to implement steps 2 and 3 jointly, by sequentially identifying increasing sets of variables that create association rules satisfying predefined minimum confidence and support requirements. The association rules in Fig. 11 shows associations that hold with confidence 0.8 and support 0.2. AutoPrognosis uses an RPy2-based wrapper to implement the CBA algorithm through the R package arulesCBA.

## Electronic supplementary material

Supplementary material
