# Supplementary material for: Prognostication and Risk Factors for Cystic Fibrosis via Automated Machine Learning
Source: Sci Rep. 2018 Jul 26;8:11242. doi: 10.1038/s41598-018-29523-2 (PMC6062529; doi:10.1038/s41598-018-29523-2)
Supplement: Supplementary file 1 — Supplementary material [file 41598_2018_29523_MOESM1_ESM.pdf]

Prognostication and Risk Factors for Cystic Fibrosis via Automated Machine Learning

Ahmed M. Alaa[1] **and Mihaela van der Schaar**[1,2,3]

[1]Department of Electrical Engineering, University of California, Los Angeles, CA, 90095, USA
[2]Engineering Science Department, University of Oxford, Oxford, OX1 3PJ, UK
[3]Alan Turing Institute, London, NW1 2DB, UK
[*]ahmedmalaa@ucla.edu

ABSTRACT

Table S1

Variable	F_1 score
Oxygen Therapy	0.5 ± 0.04
FEV1	0.47 ± 0.03
FEV1 % Predicted	0.44 ± 0.02
FEV1 % Predicted (2011)	0.44 ± 0.04
CFRD	0.42 ± 0.01
Diabetes	0.38 ± 0.02
Heterozygous	0.38 ± 0.04
Homozygous	0.37 ± 0.02
IV Antibiotic Days in Hospital	0.34 ± 0.04
FEV1 % Predicted (2010)	0.34 ± 0.05
FEV1 % Predicted (2009)	0.34 ± 0.06
Oral Corticosteroids	0.32 ± 0.02
Best FEV1	0.30 ± 0.01
Noninvasive Ventilation	0.28 ± 0.02
FEV1 % Predicted (2008)	0.27 ± 0.03
IV Antibiotic Days at Home	0.24 ± 0.04
Weight	0.21 ± 0.06
Best FEV1 % Predicted	0.21 ± 0.07
Height	0.20 ± 0.06
Non-IV Hospital Admission	0.19 ± 0.02
Age	0.19 ± 0.03

Table 1. F_1 scores achieved by individual variables.

Table S2

Variable	AUC-PR
Oxygen Therapy	0.48 ± 0.01
FEV1	0.42 ± 0.01
FEV1 % Predicted	0.41 ± 0.02
FEV1 % Predicted (2011)	0.41 ± 0.03
CFRD	0.37 ± 0.02
Diabetes	0.35 ± 0.02
Heterozygous	0.33 ± 0.01
Homozygous	0.33 ± 0.01
IV Antibiotic Days in Hospital	0.33 ± 0.02
FEV1 % Predicted (2010)	0.31 ± 0.02
FEV1 % Predicted (2009)	0.30 ± 0.03
Oral Corticosteroids	0.30 ± 0.04
Best FEV1	0.30 ± 0.05
Noninvasive Ventilation	0.29 ± 0.03
FEV1 % Predicted (2008)	0.27 ± 0.04
IV Antibiotic Days at Home	0.24 ± 0.01
Weight	0.21 ± 0.01
Best FEV1 % Predicted	0.19 ± 0.01
Height	0.18 ± 0.01
Non-IV Hospital Admission	0.13 ± 0.01
Age	0.11 ± 0.01

Table 2. Variable importance analysis after including patients lost to follow-up.

Table S3

Variable	Score
FEV1 % Predicted	1
Oxygen Therapy	0.83
IV Antibiotic Days in Hospital	0.71
Oral Corticosteroids	0.69
Noninvasive Ventilation	0.65
IV Antibiotic Days at Home	0.5
Weight	0.45
Best FEV1 % Predicted	0.41
Height	0.40
Non-IV Hospital Admission	0.34
Age	0.32
FEV1	0.23
FEV1 % Predicted (2011)	0.21
CFRD	0.15
Diabetes	0.12
Heterozygous	0.10
Homozygous	0.10
FEV1 % Predicted (2010)	0.05
FEV1 % Predicted (2009)	0.03
Best FEV1	0.02
FEV1 % Predicted (2008)	0.02

Table 3. Variable importance ranked using (normalized) correlation-based feature selection scores.
